# Update on vitamin D role in severe infections and sepsis

**DOI:** 10.1186/s44158-024-00139-5

**Published:** 2024-01-23

**Authors:** Salvatore Lucio Cutuli, Elena Sancho Ferrando, Fabiola Cammarota, Emanuele Franchini, Alessandro Caroli, Gianmarco Lombardi, Eloisa Sofia Tanzarella, Domenico Luca Grieco, Massimo Antonelli, Gennaro De Pascale

**Affiliations:** 1 Department of Emergency, Intensive Care Medicine and Anesthesia, Fondazione Policlinico Universitario A. Gemelli IRCCS, Rome, Italy; 2 Istituto Di Anestesiologia E Rianimazione, Università Cattolica del Sacro Cuore, Rome, Italy; 3 Medical Intensive Care Unit, Hospital Clinic Barcelona, Barcelona, Spain

**Keywords:** Infection, Sepsis, Septic shock, Vitamin D, Critical care, ICU

## Abstract

Severe infections frequently require admission to the intensive care unit and cause life-threatening complications in critically ill patients. In this setting, severe infections are acknowledged as prerequisites for the development of sepsis, whose pathophysiology implies a dysregulated host response to pathogens, leading to disability and mortality worldwide.

Vitamin D is a secosteroid hormone that plays a pivotal role to maintain immune system homeostasis, which is of paramount importance to resolve infection and modulate the burden of sepsis. Specifically, vitamin D deficiency has been widely reported in critically ill patients and represents a risk factor for the development of severe infections, sepsis and worse clinical outcomes. Several studies have demonstrated the feasibility, safety and effectiveness of vitamin D supplementation strategies to improve vitamin D body content, but conflictual results support its benefit in general populations of critically ill patients. In contrast, small randomised clinical trials reported that vitamin D supplementation may improve host-defence to pathogen invasion via the production of cathelicidin and specific cytokines. Nonetheless, no large scale investigations have been designed to specifically assess the impact of vitamin D supplementation on the outcome of critically ill septic patients admitted to the intensive care unit.

## Background

Sepsis is a leading cause of disability and mortality worldwide, thus representing a global challenge and a research priority for clinicians and health care systems [1]. The pathophysiology of sepsis implies a dysregulated host response to severe infection leading to multi-organ dysfunction and worse clinical outcomes [2]. The 2021 Surviving Sepsis Campaign guidelines [3] recommended the timely implementation of several interventions with the aim to lighten the burden of sepsis, which include early patient recognition, identification and effective control of the infective source, appropriate antimicrobial therapy and adequate multi-organ support. However, no therapies directly targeting the immune system dysfunction [4–8] have been suggested beyond the systemic administration of corticosteroids in specific clinical settings [9–13].

In this context, an increasing amount of research revealed the theoretic role of vitamin D to restore the immune system homeostasis and potentially limiting the consequences of inflammatory dysregulation [14–16]. Vitamin D is a secosteroid hormone, whose metabolism relies on diet, sun exposure, and liver and kidney functions, being altered by acute or chronic diseases affecting these organs [15]. Epidemiologic reports highlighted the wide diffusion of vitamin D deficiency in the community [17, 18] and its large prevalence (about 70%) among critically ill patients admitted to the intensive care unit (ICU) [19], for whom it represents a risk factor for the development of severe infections, sepsis and worse clinical outcomes [20–23]. In order to overcome these issues, several researches have demonstrated the feasibility and safety of vitamin D supplementation in critical care, which was shown effective to improve vitamin D body content [24, 25]. However, controversial results do not support its benefit on patient prognosis [26–29].

In this narrative review, we report the epidemiology of infections in ICU patients and describe the pathophysiology of sepsis. Moreover, we discuss the physiology of vitamin D, the clinical implications of vitamin D deficiency and the effect of vitamin D supplementation in critically ill patients with severe infections and sepsis. Finally, we provide an overview of ongoing clinical investigations in this field.

## Main text

### Severe infection, host response and sepsis

#### Epidemiology of infections and sepsis in ICU

Severe infections are leading causes of admission to the ICU and represent frequent complications during the ICU stay, being acknowledged as prerequisites for the development of sepsis [30]. Vincent et al. designed a 24-h point prevalence study involving 1150 centres in 88 countries with the aim to report the prevalence of infections in critically ill patients admitted to the ICU. Among 15,202 patients included, 8135 (54%) had suspected or proven infections, 1760 (22%) of whom were acquired in the ICU and represented a predictor of higher mortality compared with the community-acquired ones. Pathogen identification was reported in 5259 (65%) patients and was characterised by gram-negative bacteria (3540 patients, 67%), gram-positive bacteria (1946 patients, 37%), fungi (864 patients, 16%), viruses (196 patients, 3.7%) and parasites (43 patients, 0.8%). In comparison with previous investigations, this study reported an increasing rate of infections, which was 45% in 1992 (the EPIC I study [31]) and 51% in 2007 (the EPIC II study [32]). This finding was explained by the authors as possibly due to improvement in surveillance programmes for the early diagnosis of infection in ICU patients. Unfortunately, this study did not provide specific information on the epidemiologic and clinical characteristics of sepsis, that was reported on a large scale by the ICON study in 2012 [33]. In this paper, Vincent et al. included 10,069 patients from 730 centres in 84 countries and found that sepsis was diagnosed in 2973 (20.5%) patients on admission or during the ICU stay [33, 34]. In comparison with the SOAP study in 2002 [35], the proportion of patients with sepsis was slightly higher in the ICON study (29.6% vs 31.9%, *p* = 0.03) and characterised by increased disease severity (SAPS II score 41.9 ± 18.2 vs. 36.5 ± 17.1; SOFA scores on admission 6.3 ± 4.3 vs. 5.1 ± 3.8, maximum SOFA scores during the ICU stay 7.8 ± 4.8 vs. 6.6 ± 4.4, all *p* < 0.001), although the adjusted odds of ICU mortality was lower [OR 0.45 (0.35–0.59), *p* < 0.001] [36]. Over the last decade, the reported incidence of sepsis is increasing [37], likely due to the ageing population and greater recognition, but the true incidence remains unknown [2].

#### Inflammatory system dysfunction and sepsis

Sepsis results from the dysregulated inflammatory response to pathogens invading sterile organs or altering microbiota homeostasis with shift from symbiosis to dysbiosis [38]. Pathogen-associated molecular patterns (PAMPs) and danger-associated molecular patterns (DAMPs) are recognised by specific receptors (e.g. the toll-like receptor, TLR), whose activation induces multiple intracellular pathways leading to the expression of specific genes, which codify for inflammatory (e.g. cytokines) and metabolic molecules (e.g. hormones) [5]. The physiological rationale of immune activation triggered by pathogen recognition aims to control such a microbial threat and is counterbalanced by immunosuppressive pathways, in order to limit tissue damage. In this setting, sepsis occurs when the balance between immune activation and immune suppression is lost, and causes both metabolic derangements and organ dysfunction [2]. Traditionally, immune activation was considered the early stage of inflammation and implied the release of tumour necrosis factor-α (TNF-α), several interleukins (e.g. IL-1β, IL-2, IL-6, IL-8) and interferon-γ (IFN-γ). In contrast, immune suppression was considered the late stage of inflammation and was mediated by the release of specific molecules (e.g. IL-10). However, recent evidence demonstrated that immune activation and immune suppression coexist during the whole inflammatory response process [39] and no specific therapies have been demonstrated effective to improve the immune system dysfunction [4–8]. In this setting, an increasing amount of research have shed light on the potential of vitamin D to mitigate hyperinflammation as well as foster host defence towards infections via the enhanced production of cathelicidins.

### Physiology of vitamin D

#### Metabolism

The vitamin D family includes several lipophilic hormones characterised by a secosteroid structure [40], among those vitamin D_2_ (ergocalciferol) and vitamin D_3_ (cholecalciferol) exert the most biologically powerful activity. The intake of vitamin D_2_ comes mostly from diet (e.g. vegetables), while vitamin D_3_ is mostly produced in the skin by the reaction with solar ultraviolet B radiation, although it is contained in few aliments (e.g. animal-based food like fatty fish and egg) [41]. For these reasons, vitamin D production is influenced by sun exposure (e.g. season, latitude, clothing), diet and age (skin structure change with dermis reduction) [16].

Vitamin D is turned by the 25-hydroxylase of the liver (cytochrome p450, CYP2R1—endoplasmic reticulum) into the 25-hydroxyvitamin D [25(OH)D, calcidiol], which is further converted by the 1α-hydroxylase (cytochrome p450, CYP27B1- mitochondria) of the renal tubular cells into the most biologically active 1,25-dihydroxyvitamin D [1,25(OH)_2_D, calcitriol] [41]. Of note, the 1α-hydroxylase has been identified in several extra-renal tissues (e.g. immune and inflammatory cells), where it is supposed to contribute to intracrine and paracrine regulation pathways [42]. Vitamin D circulates into the bloodstream predominantly carried by the glycoprotein vitamin D binding protein (VDBP) [43] in a very stable complex. In contrast, free and albumin-bound compounds roughly accounts for the 10% of circulating vitamin D and are considered rapidly available for biological functions [44]. Although the 1,25-dihydroxyvitamin D is the most biologically active form of this hormone [45], the 25-dihydroxyvitamin D is characterised by greater blood level (1000 times) and longer half-life (4 h vs 2–3 weeks, respectively) [46].

#### Pathophysiologic implications

**Fig. 1:**
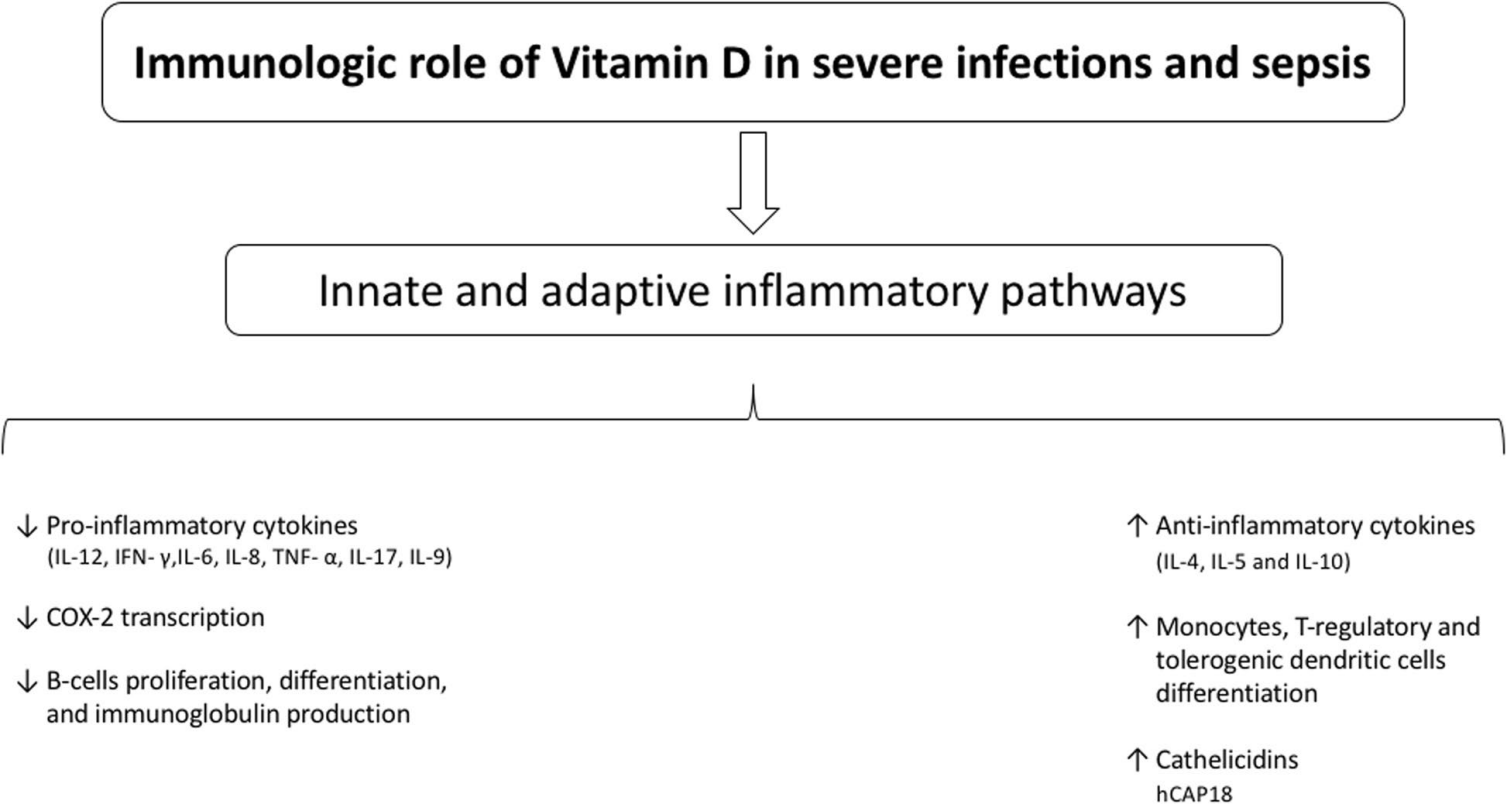
Immunologic role of vitamin D in severe infections and sepsis

Vitamin D is characterised by several pleiotropic activities beyond the calcium/phosphate balance (classical metabolic pathway), which include microbial clearance, immunomodulation of innate and adaptive responses [47], anti-tumour and cardiovascular homeostasis [15]. Over the last decades, an increasing amount of evidence has reported an association between vitamin D status alterations and inflammatory diseases [16, 48]. Specifically, 1,25(OH)_2_D influences both innate (pathogen recognition and antigen presentation) and adaptive (T and B lymphocyte function) inflammatory pathways (Fig. 1) via the nuclear vitamin D receptor (nVDR), that acts as a transcription factor modulating inflammatory cells activation, differentiation and production of cytokines [16]. The 1,25(OH)_2_D reduces the production of proinflammatory cytokines (IL-12, IFN- γ, IL-6, IL-8, TNF-α, IL-17, IL-9), increases the production of anti-inflammatory cytokines (IL-4, IL-5 and IL-10), enhances the differentiation of T regulatory cells, tolerogenic dendritic cells and monocytes to macrophages [16, 49–52]. Moreover, the 1,25(OH)_2_D inhibits the COX-2 transcription [53]. Similarly, the 1,25(OH)_2_D exerts inhibitory effects on B-cells proliferation, differentiation to plasma cells, and immunoglobulin production, while it induces apoptosis of these cells [16, 54–57], thus implying a role for vitamin D to promote immune tolerance [48]. Also, the 1,25 (OH)_2_D exerts non-genomic effects via the membrane vitamin D receptor (mVDR), thus modulating intracellular signalling pathways [16]. In severe infections and sepsis, the Toll-like receptors (TLRs) play roles of paramount importance to early recognise and rapidly respond to pathogen invasion via the activation of several inflammatory pathways [58]. The activation of the TLRs influences the production of immunological peptides that are actively involved in the host response to infection, like cathelicidins [59]. Specifically, cathelicidins are antimicrobial peptides forming α-helices that have been identified in many mammalian species, whose C-terminal domain has antimicrobial properties by both disrupting pathogen membrane and improving immune cell signalling [60]. In humans, the sole cathelicidin found was the hCAP18, which was isolated in neutrophils, monocytes, lymphocytes and epithelial cells at the barrier level [24]. The C-terminal domain of the hCAP18, the LL-37, was demonstrated to exert potent broad-spectrum antimicrobial activities against viruses and bacteria [61–64]. In critically ill patients with sepsis, the 25-hydroxyvitamin D blood concentration was demonstrated to be directly associated with LL-37 [65]. Pre-clinical investigations showed that airway epithelial cells express both the VDR and the 1α-hydroxylase [61], thus inducing the production of antimicrobial peptides like cathelicidin and defensin β4.

### Vitamin D status alterations

The 25(OH)D is the major circulating vitamin D metabolite [66] and its concentration has been widely used to assess the vitamin D status. Specifically, the Institute of Medicine [67] graded individual vitamin D status as:

- Sufficiency: 25(OH)D ≥ 30 ng/ml
- Insufficiency: 25(OH)D = 21–29 ng/ml
- Deficiency: 25(OH)D ≤ 20 ng/ml

In this context, a cut-off of 25(OH)D ≤ 12 ng/ml is considered the threshold for severe vitamin D deficiency [68] and a post-hoc analysis of the VITdAL-ICU trial [26] reported lower hospital mortality among critically with severe vitamin D deficiency who received vitamin D supplementation compared with placebo. The results of the VITdAL-ICU trial [26] will be discussed further on this manuscript.

The vitamin D deficiency is widely diffused among Western communities (40% of the subjects) [17, 18, 46], and seems to be prevalent in lower-middle income countries according to spare evidence [69], although the global epidemiology of this condition has never been investigated.

A comprehensive analysis of risk factors for vitamin D deficiency was presented elsewhere [15]. Critically ill patients represent a subgroup of the population particularly vulnerable to vitamin D deficiency, which has been reported in 40–70% of patients at the admission to the ICU [19]. In this setting, vitamin D deficiency is commonly diagnosed in patients with infection and sepsis, although the nature of this association is poorly understood and does not imply causation.

#### Severe infections and sepsis

In severe hospitalised patients, vitamin D deficiency represents a risk factor for the development of sepsis and a predictor of higher mortality [70–73]. Specifically, Moromizato et al. [74] observed that the 25(OH)D blood levels ≤ 15 ng/mL before hospital admission were predictive for the risk of sepsis and 90-day mortality in 3386 patients. In 2016, De Pascale et al. [75] conducted an observational study including 107 critically ill adults with sepsis reported Vitamin D deficiency in 93.5% of the patients, of which 53.3% showed extremely low 25(OH)D bloodstream concentration (≤ 7 ng/mL) [75]. Specifically, extremely low 25(OH)D bloodstream concentration and higher mean SAPS 2 score were independent predictors of sepsis-related mortality. Moreover, patients with extremely low 25(OH)D bloodstream concentration had a higher rate of microbiologically confirmed infections (80.7% vs 58%, *p* = 0.02) and a lower percentage of microbiological eradication (35.3% vs 68%, *p* 0.03) compared with those with 25(OH)D > 7 ng/ml [75]. Furthermore, patients with extremely low 25(OH)D bloodstream concentration required a longer duration of mechanical ventilation when affected by pneumonia, and vasopressor administration for septic shock, in comparison with those with compared with those with 25(OH)D > 7 ng/ml [75]. In 2018, Zhou et al. conducted a systematic review and a meta-analysis [76] of 24 studies to explore the association between vitamin D levels and sepsis. The authors found that the vitamin D level was lower in patients with sepsis compared to those without sepsis, but vitamin D deficiency had no significant association with sepsis-related death. Conversely, a recent systematic review and meta-analysis [77] of 8 studies (1736 patients) found that lower 25(OH)D levels were independently associated with a higher risk of mortality in patients with sepsis (adjusted relative risk: 1.93, *p* < 0.001). Subgroup analyses showed that only severe vitamin D deficiency, defined as 25(OH)D < 10 ng/ml, was significantly associated with increased risk of death in sepsis, while this relationship was not reproduced for higher 25(OH)D level [77]. Further research is warranted to clarify whether vitamin D deficiency is involved in the pathophysiology of severe infections and sepsis or should be acknowledged as a marker of patient severity.

#### Coronavirus disease-19

The coronavirus disease 19 (COVID-19) is a severe clinical condition caused by the severe acute respiratory syndrome coronavirus 2 (SARS-CoV-2), whose pathophysiology has not been completely understood and mostly relies on the immune system dysfunction caused by the SARS-CoV-2 infection [78].

In this context, an observational study on 191,779 patients found that SARS-CoV-2 positivity was higher among patients with vitamin D deficiency and inversely associated with circulating 25(OH)D levels, independently of latitude, ethnicity, age and sex [79]. A possible explanation of these findings relies on the immunomodulating function of vitamin D, especially for what concerns hyperinflammatory cytokines like TNF, whose soluble receptor was demonstrated as an independent predictor of 30-day mortality in COVID-19 patients [80]. Nevertheless, the pathophysiological implication of vitamin D deficiency in COVID-19 patients remains unknown and warrants to be clarified by future investigations.

## Rationale and clinical evidence of vitamin D supplementation in ICU

Vitamin D compounds are available for enteral, parenteral and intramuscular routes of administration. Enteral formulations have been widely used in clinical trials, being characterised by ease of administration and large vitamin D bioavailability. Specifically, some studies reported a greater increase of 25-dihydroxyvitamin D blood level in patients who received oral formulations, compared with those who received the same dose of vitamin D-based compound by intramuscular administration [81]. Parenteral and intramuscular administrations of vitamin D-based compounds should be indicated for patients with malabsorption due to enteral diseases, gastrointestinal bypass surgery and medications that reduce lipid absorption. In clinical practice, vitamin D_2_ and vitamin D_3_ represent the most widely used isoforms of this molecule and may plausibly be considered “native vitamin D”. Although both compounds are characterised by low stability in moist air [82], vitamin D_3_ administration was associated with a greater increase of 25-hydroxyvitamin D blood level, compared to vitamin D_2_ [83]. In recent years, several vitamin D analogues have been manufactured with the aim to produce specific compounds with enhanced pharmacokinetic and pharmacodynamic properties. As an example, paricalcitol does not require enzymatic activation, doxercalciferol has a prolonged half-life and maxacalcitrol specifically acts on non-classical vitamin D-associated pathways [84, 85]. However, the effect of these drugs on vitamin D status and patient clinical outcomes has never been tested in large-scale investigations.

Vitamin D supplementation is feasible, safe and effective to improve body content of this compound within a few days in critically ill patients admitted to the ICU [24, 25]. Several observational studies reported an association between vitamin D supplementation and improved clinical outcomes in hospitalised patients with severe infection [86, 87]. A recent meta-analysis pooling the results of 9 randomised controlled trials on 1867 critically ill patients [88] found no benefit of vitamin D supplementation on the 28-day mortality compared with placebo (20.4% vs 21.7%, respectively). In contrast, Menger et al. [89] conduced a larger systematic review and meta-analysis on 2449 critically ill patients from 16 randomised controlled trials and found lower mortality in critically ill patients who received vitamin D supplementation. However, both systematic reviews and meta-analyses had important limitations that hampered the generalizability of results, mostly due to the wide degree of inhomogeneity among the studies included. The only two large scale clinical investigations were the VITdAL-ICU [26] and the VIOLET [27] trials (Table 1), whose specific peculiarities warrant to be discussed in detail.

**Table 1:** Large-scale randomised clinical trials on vitamin D supplementation in hospitalised patients

Authors, year of publication	Study sites	Study duration	Number of patients	Inclusion criteria	Intervention	Primary outcome	Patients characteristics	Main result
Amrein et al. 2014 [26]	Single centre, Austria	2012–2015	475	Adult white critically ill patients, expected length of ICU stay ≥ 48 h and with 25-hydroxyvitamin D blood level of 20 ≤ ng/mL	Enteral vitamin D3 protocol administration: 540,000 IUs followed by monthly 90,000 IU for 5 months Vs Placebo	Length of hospital stay	Surgical patients were prevalent Severe infections/sepsis: ~ 8% at admission	No difference for the primary outcome
Ginde et al. 2019 [27]	44 centres, USA	2017–2018	1078	Adult patients with with > 1 risk factors for death or lung injury, deemed to be managed in the ICU and with 25-hydroxyvitamin D blood level ≤ 20 ng/mL	Enteral vitamin D3 protocol administration: 540,000 IUs Vs Placebo	90-day mortality rate	Medical patients were prevalent Severe infections/sepsis: ~ 33%	No difference for the primary outcome
Murai et al. 2021 [29]	2 centres, Brazil	2020	240	Adult patients with moderate to severe COVID-19	Oral vitamin D_3_ protocol administration: 200,000 IUs Vs Placebo	Length of hospital stay	Severe infections/sepsis: not declared	No difference for the primary outcome
Mariani et al. 2022 [90]	17 centres, Argentina	2020–2021	218	Adult patients admitted to general ward in the last 24 h with mild-to-moderate COVID-19 and risk factors for disease progression	Oral vitamin D_3_: 500,000 IUs Vs Placebo	Change in the respiratory SOFA between baseline and the highest rSOFA recorded up to day 7	Severe infections/sepsis: not declared	No difference for the primary outcome

*Abbreviations*: *SOFA* Sepsis-related Organ Failure Assessment

The VITdAL-ICU trial [26] was conducted in 5 ICUs of an Austrian hospital and enrolled 475 critically ill patients with vitamin D deficiency, who were randomised to receive oral vitamin D supplementation (vitamin D_3_: loading dose of 540,000 IU, followed by monthly maintenance dose of 90,000 IU for 5 months) or placebo. In patients receiving vitamin D supplementation the deficiency status improved within 7 days from the inclusion and remained stable for the 28 days afterwards respect to the placebo. Although Vitamin D supplementation had no effect on patient clinical outcomes, a subgroup analysis including patients with severe vitamin D deficiency [25(OH)D levels ≤ 12 ng/mL)] showed that vitamin D supplementation reduced the risk of hospital mortality compared with placebo (28.6% vs 46.1%, respectively). In the light of this finding, the VIOLET trial [27] enrolled 1358 severe patients with vitamin D deficiency from 44 hospitals in the United States, who were randomised to receive enteral vitamin D supplementation (vitamin D_3_: loading dose of 540,000 IU, administered even before ICU admission and not followed by maintenance dose) or placebo. Although vitamin D status improved in patients who received vitamin D supplementation compared with placebo, this intervention had no impact on patient clinical outcomes. Accordingly, this study was stopped for futility after the first interim analysis. Unfortunately, the prevalence of sepsis in the VITdAL-ICU [26] and VIOLET [27] trials was low (7.7% and 33.3%), thus impairing to evaluation of the effect of vitamin D supplementation in this patient population.

During the COVID-19 pandemic, Murai et al. [29] investigated the effect of vitamin D_3_ supplementation (single dose: 200,000 IU) vs placebo in 240 hospitalised patients (most of whom were not critically ill) with moderate to severe COVID-19 from 2 centres and found no difference of hospital length of stay between study groups. In this setting, Mariani et al. [90] randomised 218 hospitalised patients with mild-to-moderate COVID-19 and risk factors for disease progression from 17 centres to receive vitamin D_3_ (single dose: 500,000 IU) vs placebo and found no difference in terms of respiratory SOFA score changes between the baseline and the highest value within the following 7 days. In both of these studies, the increase of vitamin D blood levels in patients who received vitamin D supplementation compared to placebo did not correspond to any improvement of secondary outcomes as ICU admission, need for mechanical ventilation, and hospital mortality.

In contrast, Leaf et al. randomised 67 critically ill patients with sepsis to receive 2 μg of intravenous calcitriol or placebo [28], with the aim to investigate whether vitamin D supplementation may improve cathelicidin blood levels within 24 h (primary outcome), increase 1,25-dihydroxyvitamin D blood level within 6 h, influence cytokines mRNA expression and cytokines levels into the bloodstream within 24 h and reduce urinary markers of kidney injury within 48 h (secondary outcomes). Although vitamin D blood levels increased in patients who received vitamin D supplementation, it was only associated with an increase of cathelicidin and IL-10 mRNA expression within 24 h compared with placebo. Vitamin D supplementation was not associated with 28-day, ICU and hospital mortality compared with placebo, whereas this trial was not powered to assess these outcomes. Moreover, Quraishi et al. randomised 30 critically ill septic patients to receive enteral vitamin D_3_ 400,000 IU versus of vitamin D_3_ 200,000 IU versus placebo. They found that the administration of higher doses of vitamin D_3_ led to an improvement of 25(OH)D body content and bioavailability at day 5, which corresponded with a concomitant increase of LL-37 [24].

## Future directions

The VITDALIZE study (NCT03188796) [66] is an international (Austria, Germany, Belgium, Switzerland and UK), multicentre (more than 30 sites), placebo-controlled, double-blind, phase 3 randomised trial, that has started in 2017 and aims to include 2400 adult critically ill patients with severe vitamin D deficiency, within the first 72 h of ICU admission. Patients randomised in the treatment group receive vitamin D_3_ 540,000 IU at the enrolment, followed by 4000 IU daily for 90 days. The primary outcome of the study is 28-day mortality, while the development of infections requiring antibiotics at day 90 is accounted among other secondary outcomes (e.g. progression of organ dysfunction, hospital, ICU, 90-day and 1-year mortality).

Conversely, there are no researches recorded in *ClinicalTrial.gov* currently ongoing with the aim to assess the effect of vitamin D supplementation to prevent the development of infection or sepsis as well as improve the outcome of critically ill patients suffering from these conditions. Due to the clinical relevance of vitamin D deficiency and the potential of vitamin D supplementation to improve the outcome of critically ill patients with severe infections and sepsis, future multicentre clinical trials in this context appear clinically justified and urgently advocated.

## Conclusion

Severe infections and sepsis are widely diagnosed in ICU patients, for whom vitamin D deficiency is frequent and associated with the development of these conditions. Vitamin D was demonstrated to modulate the immune system and vitamin D deficiency may represent a target for future therapy, in order to control infection and ameliorate inflammatory dysfunction and sepsis. Although small randomised clinical trials demonstrated a benefit of vitamin D supplementation to improve immunologic features of in critically ill septic patients, no large scale randomised controlled trials have been designed to specifically assess the effect of this therapy on patient-related clinical outcomes. Accordingly, future high-quality research is urgently advocated in this field.

## Data Availability

Not applicable.

## Abbreviations

COVID-19: Coronavirus disease 19
DAMPs: Danger-associated molecular patterns
25(OH)D: 25-Hydroxyvitamin D
1,25(OH)_2_D: 1,25-Dihydroxyvitamin D
IFN-γ: Interferon-γ
ILs: Interleukins
ICU: Intensive care unit
IU: International unit
nVDR: Nuclear vitamin D receptor
PAMPs: Pathogen-associated molecular patterns
SOFA: Sequential Organ Failure Assessment
SARS-CoV-2: Severe acute respiratory syndrome coronavirus 2
SAPS II: Simplified Acute Physiology Score II
TLR: Toll-like receptor
TNF-α: Tumour necrosis factor-α
VDBP: Vitamin D binding protein
